# An Investigation of Language-Specific and Orthographic Effects in L2 Arabic geminate production by Advanced Japanese- and English-speaking learners

**DOI:** 10.1177/00238309241267876

**Published:** 2024-08-30

**Authors:** Albandary Aldossari, Ryan Andrew Stevenson, Yasaman Rafat

**Affiliations:** Department of Psychology, The University of Western Ontario, Canada; Brain and Mind Institute, The University of Western Ontario, Canada; Western Institute for Neuroscience, The University of Western Ontario, Canada; Program in Neuroscience, The University of Western Ontario, Canada; Department of Language and Cultures, Faculty of Arts and Humanities, The University of Western Ontario, Canada; Brain and Mind Institute, The University of Western Ontario, Canada

**Keywords:** L2 speech learning, production, geminate, diacritics, Japanese, Arabic, English

## Abstract

Research has indicated that second-language learners have difficulty producing geminates accurately. Previous studies have also shown an effect of orthography on second-language speech production. We tested whether the existence of a contrast in the first language phonology for length aids the second-language production of the same contrast. Furthermore, we examined the effect of exposure to orthographic input on geminate consonant production in a cross-script context. We tested the production of Arabic geminate-singleton stop consonants [/bː/-/b/, /tː/-/t/, /dː/-/d/, and /kː/-/k/], a nasal stop consonant /mː/-/m/, and an emphatic stop consonant /tˤː/-/tˤ/, as well as the effect of the diacritic used in Arabic to mark gemination in a delayed imitation task and two reading tasks (ortho-with diacritics and ortho-without diacritics). A comparison of the productions of advanced Japanese-speaking learners, English-speaking learners, and an Arabic control group showed that both learner groups were able to produce Arabic geminate stops; however, the Japanese-speaking learners exhibited an advantage over the English-speaking learners in the auditory-only task and in the presence of diacritics, highlighting the fact that orthographic effects may occur in some cross-script contexts.

## 1 Introduction

The objectives of this study are twofold. First, this study seeks to determine whether the presence of a phonological distinction in the first language (L1) leads to the production of a similar distinction in the second language (L2) that is comparable to the target production (i.e., L2). Specifically, it examines whether Arabic geminate stop consonant production is more difficult for English-speaking learners (ELs) than Japanese-speaking learners (JLs). Gemination is contrastive in Japanese (Fujisaki et al., 1975; Fujisaki & Sugito, 1977; Warner & Arai, 2001) and Arabic (^Abu Guba, 2021, 2023;^ Ahmad, 2012; Almutiri, 2015; Al-Tamimi, 2004) but is phonetic in English and occurs at word boundaries (Ladefoged, 1999; Roach, 2000; Wolfram & Schilling-Estes, 1998). Moreover, geminate-singleton ratios are larger in Japanese (Beckman, 1982; M. S. Han, 1962, 1992; Homma, 1981) than in Arabic (Ahmad, 2012).

Several studies have examined L2 geminates with regard to production (Almutiri, 2015; Bassetti et al., 2018; M. S. Han, 1992; Harada, 2006; Mah & Archibald, 2003; Rafat, 2015, 2016; Sokolović-Perović et al., 2020; Young-Scholten, 2004) and perception (Celata & Cancila, 2010; Hayes-Harb, 2005), showing that they are difficult to acquire for learners who do not have a length contrast in their L1. Although there is abundant evidence to show that geminates are either hypoarticulated (M. S. Han, 1992; Sorianello, 2014) or hyperarticulated (Harada, 2006; Mah & Archibald, 2003) by L2 learners, we are unaware of any studies examining L2 geminate production by learners whose L1 has a phonological length contrast, such as Japanese.

Flege’s Speech Learning Model (Flege, 1995) predicted that the degree of acoustic-phonetic distance predicts whether an L2 sound can be acquired. In other words, “old/existing” sounds are not problematic, “new” sounds are easy to acquire, but “similar” sounds are difficult to acquire. Flege and Bohn’s (2021) Revised Speech Learning Model (SLM-r) proposes that it is the degree of perceived phonetic dissimilarity between L1 and L2 sounds which determines how easily/well a sound can be acquired. In other words, it predicts that the smaller the perceived phonetic distance between L1 and L2 sounds, the more difficult the acquisition of L2 sounds. The SLM-r also assumes the full access hypothesis, which predicts that if a new phonetic category is formed for an L2 sound, it may differ from that of the native speakers’ if the L2 sound is defined, at least partially, by features not used in the learner’s L1. Given that geminates do not exist in English intervocalically, the SLM-r would predict that ELs may not perceive the phonetic distance between them and their L1 singletons as vast enough, thus producing them with difficulty. In other words, the ELs may categorize geminates as singletons. On the other hand, if ELs form a new category for geminates, these categories of geminate and singleton may still differ from those of the native speakers. However, geminates should not be difficult for JLs because they already exist in the L1.

The second aim of the study is to explore the effect of orthographic input on geminate stop production in L2 Arabic. Specifically, we examine the effect of exposure to diacritics in Arabic script. Previous studies have shown that orthography can affect L2 speech production (Bassetti, 2017; Bassetti et al., 2015, 2018; Hayes-Harb & Barrios, 2021; Rafat, 2015, 2016; Rafat & Stevenson, 2018). In particular, orthographic input my modify the effects of auditory input and promote (e.g., Bassetti et al., 2015; Rafat, 2015), or hinder (e.g., Bassetti, 2017; Bassetti et al., 2015; Rafat, 2015, 2016; Rafat & Stevenson, 2018) target-like production, or have no effect when acquisition is more strongly based on oral communication (Mitterer, 2021). Moreover, more recently, it has been argued that the role of orthography can be superseded by higher prosodic constraints (Abu Guba, 2021, 2023). Although the literature on auditory-orthographic interaction in L2 speech learning has been growing, little is known about the effect of diacritics (Showalter and Hayes-Harb, 2013). In addition, orthography may affect learners whose L1 has a different script (^Rafat et al., 2021^; Sokolović-Perović et al., 2020).

The research questions of this study are as follows:

1. Is there an effect of L1 phonology on the L2 production of Arabic geminate consonants? Specifically, will both JLs and ELs be able to produce L2 Arabic geminate consonants?
2. Is there an effect of exposure to orthographic input? Specifically, is there a difference between Arabic geminate consonant production when L2 learners imitate Arabic geminates without exposure to orthographic input, read Arabic geminates without diacritics, or read Arabic geminates with diacritics?

## 2 Geminates in Arabic, Japanese, and English

Geminates are found in only 3.3% of world languages (Maddieson, 1984). Length is phonemic in some languages such as Italian, Japanese, Arabic, Hebrew, Finnish, Farsi, and Hungarian. Consonantal length contrasts are long sounds and may phonologically contrast with their short counterparts (Ladefoged & Johnson, 2006). Phonetically, the most salient acoustic cue differentiating singletons and geminates across languages is duration.

Arabic has a phonemic length contrast. For example, whereas /sɑ**kː**ɑrɑ/ means “closed,” /sɑ**k**ɑrɑ/ means “someone got drunk.” Arabic geminates occur in word-medial and final positions (Mitchell, 1990). Gemination is indicated orthographically with a diacritic called the shadda (ــّـ) (e.g., <دَرَّسَ> /ˈdɑrːɑsɑ/ “he taught someone else” versus <دَرَسَ> /ˈdɑrɑsɑ/ “he studied”). However, the geminate diacritic is used rarely and mostly in textbooks in elementary schools and religious scripts and sometimes for clarification if the meaning is ambiguous. In Arabic, diacritics represents vowel sounds and other phonetic features. The script primarily denotes consonants, whereas vowels are indicated using diacritic marks above or below consonantal letters. The mean Arabic geminate-singleton stop closure duration ratio is 2.58 (see Ahmad, 2012), where average geminates and singletons have been reported as being 171.84 and 65.6 ms long, respectively. Khattab (2007) had reported a duration range of 168–227 ms for geminates and 63–132 for singleton stops.

Similar to Arabic, Japanese has a phonological length contrast for both vowels (long vs. short) and consonants (geminate vs. singleton) (Fujisaki et al., 1975; Fujisaki & Sugito, 1977; Warner & Arai, 2001). Geminates can occur in the intervocalic positions (Tsujimura, 2017). However, not all consonants have a geminate counterpart (Kawagoe, 2015). Geminates are generally restricted to nasals and voiceless obstruents (e.g., /p t k s/) with some exceptions (^Vance, 1987).^ First, no voiced obstruent (/b, d, g/) can be geminated in the native lexicon, as found in Yamato and Sino-Japanese dialects (^Ito & Mester, 2017). For example^, /tada/ “only” changes into /tatːa/ and not /tadːa/ when geminated for emphasis. However, long voiced consonants [bː, dː, gː] can be found in recent loanwords, e.g., [bedːo] “bed,” [bagːu] “bag,” and [dogːu] “dog,” although they also tend to be devoiced, (e.g., [betːo] “bed”) (Fujimoto & Funatsu, 2018). In addition, although the native phonology of /h/ allows for gemination in various contexts, it historically alternates with [pː] rather than [hː] (e.g., /ni.hon/ “Japan” vs. /nipːon/ ‘^Japan’; Ito & Mester, 2017^).

Although geminate-singleton stop consonant ratios have been reported to be between 2.6 and 3.3 (Beckman, 1982; M. S. Han, 1962, 1992; Homma, 1981), geminate stop consonants are similar in duration to Arabic geminates, although they are a bit shorter, ranging between 134 and 183 ms (Homma, 1981). Still, Japanese singletons appear to be much shorter than Arabic singletons, ranging between 41 and 77 ms (Homma, 1981).

On the other hand, in English, gemination is phonetic (Hedia & Plag, 2017; Ladefoged, 1999; Roach, 2000; Takeuchi, 2010; Wolfram & Schilling-Estes, 1998). Moreover, unlike Arabic and Japanese, English has morphological geminates; that is, geminates in English exist word internally through afﬁxation (e.g., unnecessary) or compounding (e.g., fun name) (Hedia, 2019). Therefore, a comparison of Japanese and English is suitable for investigating the effect of L1 phonology on L2 acquisition of geminate production.

## 3 Orthographic representations in Arabic, Japanese, and English

Orthographic systems across languages have been described as shallow or deep. Shallow orthographic systems reflect surface phonology with a high level of consistency, whereas the level of consistency in languages with deep orthographies is low (Borleffs et al., 2017). Italian, Spanish, Greek, and German are examples of languages having shallow orthographies, whereas English and Hebrew are described as languages with a deep orthography (Katz & Frost, 1992; Rahbari & Lefevre, 2005; Ranbom & Connine, 2007). Shallow orthographic systems are characterized by one-to-one grapheme-to-phoneme correspondences, whereas deep orthographic systems are characterized by one-to-many or many-to-one mapping. For example, many-to-one grapheme-to-phoneme (sound) relationships are abundant in English (e.g., <f> and <gh> can both correspond to the phoneme /f/ as in <fish> [fɪʃ] and <cough> [kɒf]), but one-to-one grapheme-to-phoneme relationships prevail in Spanish (e.g., <f> corresponds only to the phoneme /f/, as in <fama> [ˈfa.ma]).

The Arabic script is classified as a consonantal alphabet, known as an Abjad (Daniels, 1992) and is written from right to left. In an Abjad, according to Saiegh-Haddad and Joshi (2014), symbols represent consonants, with the reader responsible for filling in the appropriate vowels. This writing system aligns well with the Arabic language’s root and word pattern structure, where the core semantic meaning is conveyed by consonantal roots, and vowel information can be deduced from the vocalic word pattern (Saiegh-Haddad & Joshi, 2014). Of the 28 letters in the Arabic alphabet, all except aleph represent consonants. Among these, three letters, namely /a/, /i/, or /u/, are referred to as “حروف العلة” (ħuru:f al-ʕilla), or “letters of defectiveness.” They are used to represent the three long vowels in Standard Arabic: high front /i:/, high back /u:/, and low /a:/ (Saiegh-Haddad & Joshi, 2014). The ambiguity in syllable completion is limited and can be easily resolved by a reader who is proficient in the language. In this style of writing, a deep understanding of the language is essential, as words cannot be read without the reader’s ability to provide the missing vowels (Lassegue & Longo, 2012).

Arabic words are typically based on a three-letter root, although some verbs can have four consonants (i.e., quadriliteral roots). These derivations are known as “forms,” and there are 15 recognized forms in Arabic, with the initial 10 being the most frequently used. Each form conveys a different meaning. Scholars like Ford (2009) have explained the concept of the triliteral verb root, as well as how causatives can be derived from basic verb roots. Glanville (2011) further clarified that all verb forms are third masculine singular perfective. This paper concentrates on the presentation of Forms I and II because they are the exclusive forms under examination in our study. Form I serves as the basis from which all other forms are derived. This form is characterized by the vowel in its second syllable, following the pattern (C1aC2aC3a), where the stem vowel, positioned in the base, is one of three phonemic (short) vowels. An example of this form is <kasara> C1aC2VC3a (FORM I). On the other hand, Form II “Gemination,” is distinguished from Form I by geminating the middle radical of the root, and the pattern of this form is C1aC2C3aC4a. A concrete example of this form is <fahhama> (“explaining something to someone else/made understand”).

Arabic orthography is considered deep if it is not vowelized but shallow if it is (Abu-Rabia, 1995). Diacritics are employed only in educational and religious texts for children and beginner learners to produce the target sounds, whereas advanced L2 learners can read nonvowelized written words (Hermena et al., 2015; Saiegh-Haddad & Joshi, 2014; Taha, 2016). In Arabic, diacritics are used to represent vowel sounds and gemination. The geminate diacritic is called the shadda (ــّـ). For example, in <كَتَّبَ> /kɑtːɑbɑ/, “someone asked someone else to write,” the vowels and the geminate diacritic are marked, but this is not the case in <كتب> /kɑtɑbɑ/. If this word was written with no diacritics and no semantic context, it could have four different meanings each with different pronunciations (i.e., “wrote,” “to make someone else write,” “it was written,” or “books”), which is an instance of deep orthography.

The Japanese orthographic system is both deep and shallow. Japanese is written using a logographic system (Kanji), which is imported from Chinese, and a phonographic system called Kana, which contains two syllabaries (Hiragana and Katakana; Frost, 2005). Kanji, a key component of the Japanese writing system, is not uniformly used across Japan as commonly perceived. Its application varies regionally in terms of frequency, font style, readings, and contextual use. These variations are particularly notable in proper nouns, such as place and family names, influenced by local geographical, political, and historical factors. This regional diversity in Kanji usage underscores its cultural and linguistic depth within different parts of Japan (Sasahara, 2022). Traditionally, written Japanese is read vertically top to bottom and then right to left. Thus, pages should be turned from left to right. The character-to-sound relationships in Kana can be interpreted as a one-to-one symbol-sound mapping (Dylman & Kikutani, 2018; Ijuin & Wydell, 2017; Rahbari & Lefevre, 2005). This is why Kana is considered shallow (Katz & Frost, 1992). However, Kanji is processed via the lexico-semantic route/pathways (Dylman & Kikutani, 2018; Ijuin & Wydell, 2017). That is, the relationship between Japanese Kanji characters and pronunciations is deep (Dylman & Kikutani, 2018; Hino et al., 2011; Ijuin & Wydell, 2017) and decoding takes place at the lexical/word level. The small kana/sokoun <*tsu*> (っ) is used to mark a geminate consonant in Hiragana which is used for native words (e.g., <きた> /ki**t**ɑ/ “came; arrived” but <きった> /ki**tː**ɑ/ “cut; sliced”) while the consonantal length is orthographically marked as ッ for katakana which is used for loanwords (e.g., <ベッド> /bedːo/ “bed”; Iwasaki, 2002). In contrast, consonantal length is not orthographically marked in kanji because its characters do not correspond to a single pronunciation, and it is logographic and, therefore, the symbols map to units of meaning rather than units of sound. This is often interpreted to be a deep orthography as there are no or comparatively few pronunciation cues (e.g., <切きっ手て> /kitːe/ “stamp” but <来手きて> /kite/ “someone who is coming/visitor”; ^Hino & Lupker, 1998; Ellis et al., 2004)^.

## 4 L2 geminate acquisition

Previous studies have reported that L2 learners from a variety of language backgrounds whose L1 lacks the geminate-singleton contrast struggle with L2 geminate production (e.g., Almutiri, 2015; ^Cordero et al., 2018^; M. S. Han, 1992; Harada, 2006; Hayes-Harb & Masuda, 2008; Mah & Archibald, 2003; Sorianello, 2014).

Building on this claim, the literature on L2 production provides evidence that geminates are difficult for L2 learners and are produced in a nonnative-like manner. Specifically, geminates are prone to hypoarticulation (e.g., Almutiri, 2015; M. S. Han, 1992; Hayes-Harb & Masuda, 2008; Sorianello, 2014), hyperarticulation (e.g., Harada, 2006; Mah & Archibald, 2003), or both hypo- and hyperarticulation (e.g., Cordero et al., 2018). For example, Han (1992) found that fluent American ELs of Japanese had difficulty producing geminate-singleton contrasts in a native-like manner, where the overall geminate-singleton stop closure ratio for fluent American ELs of Japanese was 1.84 on average, smaller than that of native Japanese speakers (2.83). Similar results were found with the production of Italian geminates by German-speaking, Spanish-speaking, and British-speaking learners of Italian whose proficiency level in Italian ranged between A2 and B2 according to the Common European Framework (Sorianello, 2014). Using a spontaneous conversation task in that study revealed that all learners had smaller geminate-singleton ratios than the Italian control group. Similarly, studying beginner and advanced English learners of Arabic, Almutiri (2015) showed that advanced English learners of Arabic produced longer geminate durations (172.9 ms) than beginners (159.3 ms), where the former had smaller geminate-singleton ratios than Arabic native speakers (1.76 and 3.52 for L2 learners and native speakers of Arabic, respectively). In addition, Hayes-Harb and Masuda’s (2008) study found that while inexperienced English L2 learners of Japanese were not able to produce geminates in Japanese, experienced English L2 learners of Japanese hypoarticulated the geminates in comparison with the native speakers, in a production task.

Although the latter studies provide evidence for the hypoarticulation of geminates, geminates have also been shown to be hyperarticulated. For example, Mah and Archibald (2003) found that after four months of Japanese classes, the participant, an English-speaking learner of Japanese, had larger geminate-singleton duration ratios than those of native Japanese speakers, in a reading task. In addition, Cordero et al. (2018) have provided evidence for both hypo- and hyperarticulation of geminates in the imitation of Italian geminates and Cuban Spanish geminates by native speakers of Colombian Spanish. Geminate length (the absolute duration of a segment) in Cuban Spanish was the main predictor of the type of production, where longer geminates were hypoarticulated and shorter geminates were hyper-articulated.

Together, these studies thus indicate that L2 learners whose L1 lacks a length contrast may have difficulty producing geminates in a target-like manner. Moreover, geminates are either hypo- or hyperarticulated or both hypo- and hyperarticulated by L2 learners in production. In addition, advanced learners may outperform beginner learners in geminate production, although their production may not be target-like.

## 5 Auditory-orthographic interaction in L2 speech learning

The literature on the influence of orthographic input on L2 acquisition has grown in recent years (e.g., Bassetti et al., 2015; J. Han & Choi, 2016; Mitsugi, 2018; Rafat & Stevenson, 2018; Shea, 2017; Sokolović-Perović et al., 2020; Steele, 2005). L2 speech production and perception can also be affected by orthographic input (Bassetti et al., 2015). L2 orthographic input can lead to positive effects on L2 production. Steele (2005) studied the production of stop-liquid clusters by beginning Mandarin-speaking learners of French via a word-learning task and found a positive effect of orthography. There were two groups: an orthographic group and a nonorthographic group. The results for the first group suggested that not only was the rate of rhotic deletion in voiceless clusters lower than in the second group but also there was a greater proportion of target-like voiceless stop-rhotic realizations. The results also showed that when deletion occurred, the voiceless stop-rhotic clusters were produced as single aspirated stops (e.g., target **pr**éfet /**pʁ**efε/ was produced as [pχefε] or [phefε]). Because of the phonetic similarity between aspirated stops in Mandarin and French voiceless stop-rhotic clusters, in which the rhotic tends to be realized as a voiceless fricative, Steele (2005) proposed that learners misperceive these clusters as an aspirated stop in the absence of orthography.

Similarly, the claim that exposure to orthography promotes target-like L2 production has been put forth by Rafat (2015). She examined the assibilated/fricative rhotic productions of 20 naive ELs of Spanish. The learners were assigned to two groups: auditory-only and auditory-orthographic. She found that exposure to orthography promoted a higher rate of target-like [r̆] (e.g., <ahitar> [aitar̆]) productions in naive ELs of Spanish in the production of the auditory-orthographic group than in the auditory-only group.

The limited body of research exploring the effects of diacritics on production highlights the importance of including perception-related studies in this section. To illustrate, previous studies have revealed a positive influence of diacritics on acquiring L2 phonology (e.g., Abu-Rabia, 1997; Showalter & Hayes-Harb, 2013). Showalter and Hayes-Harb (2013) examined the L2 native English perception of Mandarin tone marks. During a word learning phase, participants learned to associate Mandarin nonwords varying in lexical tone with orthographic forms (written in pinyin with/without tone marks) and pictured “meanings.” There were two conditions: a tone mark condition and a no-tone mark condition. In the first experiment, the participants listened to the auditory representation, saw both the picture and the orthographic representation with the diacritic tone mark (e.g., see <gī> and its associated picture; hear [ɡi-tone1]) and determined whether the heard form matched the accompanying picture. In the second experiment, the participants listened to the auditory form and saw the orthographic representation without tone marks (e.g., see <gi> and its associated picture; hear [ɡi-tone1]). The findings showed that the tone mark group had an above-chance performance, while the no-tone mark group had an at-chance performance. Abu Rabia (1997) had also shown a positive effect of diacritics on L2 production. The purpose of his study was to see how Arabic vowels (i.e., diacritics) and Arabic context affected the reading accuracy of both experienced and inexperienced native Arabic readers when reading narrative stories and newspaper articles. Tenth-grade native Arabic speakers grouped as inexperienced readers and experienced readers participated in the study. There were four reading conditions for each text: vowelized text, unvowelized text, vowelized word naming, and unvowelized word naming. The findings revealed that diacritics and contexts were key variables in facilitating word recognition in Arabic orthography in both inexperienced and experienced readers. Whereas some previous studies have shown that diacritics have a positive effect on acquiring L2 phonology, to our knowledge, the current study is the only one that focuses on the positive effect of L2 diacritics on two languages with two distinct scripts: English (alphabetic) and Japanese (“Kanji” logographic and “Kana” syllabic) learning Arabic which has a different script (Abjad).

Whereas the above studies have provided a positive effect of exposure to orthographic input, a number of studies have shown that exposure to orthographic input can result in nonnative-like realizations, such as sound additions (e.g., Bassetti & Atkinson, 2015; Browning, 2004), deletions (e.g., Bassetti, 2007), and substitutions of target sounds (e.g., Bassetti, 2017; Bassetti et al., 2018; Erdener & Burnham, 2005; Rafat, 2016; Rafat et al., 2021; Sokolović-Perović et al., 2020; Young-Scholten, 2004), as well as combination productions (e.g., Rafat & Stevenson, 2018).

With respect to addition of sounds, Bassetti and Atkinson (2015), for example, observed that Italian L2 learners of English produced letters that should be silent. That is, sounds can be added when they are represented in the orthographic forms of L2 words (e.g., comb /kəʊm/ is produced as *[kəʊmb]). On the other hand, L2 sounds that are not represented in the L2 orthographic forms may also be omitted by L2 speakers. For instance, Bassetti (2007) found that advanced Italian-speaking learners of Mandarin had non-target-like pronunciations when triphthongs were spelled with only two letters in pinyin (e.g., <iu> for /iou/). That is, Chinese learners omitted one vowel (producing the triphthong as a diphthong). However, their productions were accurate for the vowel /o/ in the triphthong /iou/ when it was written with three letters as in <you> .

Sounds can also be substituted in the L2. Such substitution is, in fact, the most frequently investigated orthographic effect on L2 phonology (Bassetti et al., 2020). For example, in a longitudinal study, Young-Scholten (2004) examined the production of the final obstruent devoicing rule in German by three American students who spent a year at a German secondary school. The results showed that participants had difficulty acquiring this rule. For example, they substituted the German word <*kind*> /kɪnt/ with *[kɪnd]. These results suggested that exposure to orthographic input negatively affected the acquisition of the devoicing rule of German final obstruents by these ELs.

Rafat (2016) also reported L1 substitutions as a result of exposure to orthographic input under different auditory-orthographic training and testing conditions and types of grapheme-to-phoneme correspondences on L1-based phonological transfer in L2 production in novice ELs of Spanish. Exposure to orthographic input at the time of training yielded a significantly higher rate of L1-based phonological transfer. This is in comparison to when orthographic input was presented at production or testing only, in the case of shared graphemes that correspond to different sounds in English and Spanish. Moreover, different grapheme-to-sound correspondences resulted in significantly different rates of transfer. For example, <ll> -/j/ resulted in the lowest rate of transfer (e.g., */palete/ for <pallete> /pajete/), whereas <v>-/b/ (e.g., */veneno/for <veneno> /beneno/) and <d>-/δ/ (e.g., */kodena/ for <codena> /koδena/) resulted in the highest rates of transfer in orthography under the training condition. The author proposed that a larger acoustic distance between L1 and L2 sounds in the case of shared graphemes had triggered the higher rate of transfer.

Negative effects of orthography were also found in Bassetti et al. (2018). The authors tested whether exposure to English orthography would lead to the production of English homophonic word pairs as phonological minimal pairs by Italian learners. The results of a reading-aloud task revealed that advanced learners and highly proficient bilinguals produced English homophonic word pairs as minimal pairs. For instance, <finish> /’fɪnɪʃ/ was produced as /’fɪnɪʃ/ with a singleton [n], but <Finnish> /’fɪnɪʃ/ was produced as /’fɪnːɪʃ/ with a geminate [nː]. That is, L2 learners substituted the target sound, which is a short sound, [n] with a long one [nː] because of the number of consonant letters in the spelling of the word <Finnish>. It was proposed that L1 orthographic knowledge can influence L2 phonological productions, especially when L1 L2 orthographic forms look similar. In this case, the L1 and L2 use single and double letters; however, Italian double letters represent geminates, but English double letters do not.

Moreover, Rafat et al. (2021) tested the production of the digraph <mm> in the word <summer> /sʌməɹ/ and the production of <m> in the word <stomach> /stʌmək/) by Korean- and Farsi-English bilinguals in Canada in word-naming and reading tasks. Whereas English employs a Roman alphabetic system, the Korean orthographic system uses a syllabary system called Hangeul. In Hangeul, each character, or *jamo* is arranged into blocks, which is equivalent to a single syllable. There are two copies of the consonant used to represent the geminate, although they can appear either as one “double” jamo (e.g., <도끼> /doggi/ “axe”), or separately as two jamo (e.g., <직기> /t͡ɕi**g.g**i/ “loom”). Geminates of obstruents are also represented as two repeated Latinate characters in the three most popular romanization systems for Korean. Farsi, on the other hand, uses a deep(er) orthographic system based on the Arabic orthographic system, where geminates are represented with the same diacritic as in Arabic. Moreover, digraphs are also read as geminates. The results showed that in comparison to their Farsi-English bilingual counterparts, in both the word-naming and word-reading tasks, Korean-English bilinguals substituted the target sound <m> with a longer one <mm> (e.g., <summer> /sʌmːəɹ/). This result was attributed to the orthography-induced transfer of Korean geminates into English. It was also hypothesized that the language-specific differences were due to the three following possibilities: (1) Korean is a language with a shallow writing system but Farsi has a deep(er) orthographic system, (2) transfer in Korean-English bilinguals occurred because <mm> also cues a geminate sound in the Latinized alphabetic system of Korean and (3) variability in how gemination is cued in the Farsi orthographic system blocked transfer in the Farsi-English bilinguals.

The results of Sokolović-Perović et al.’s (2020) study also provide evidence for L1 substitution. In their study, they examined whether orthographic input had an effect on L2 English productions by Japanese native speakers. Their participants were Japanese-English bilinguals and British-English native speakers who performed a delayed word repetition task and a spelling task. Each target sound was spelled either as a singleton (/t/ as in city) or as a geminate (/t**ː**/ as in kitty). The findings revealed that bilinguals substituted the target English sounds depending on the number of graphemes. That is, L2 learners produced the same English consonant as longer when it was spelled with two letters (e.g., [t**ː**] in kitty) than one (e.g., in [t] city).

There is evidence to suggest that exposure to digraphs may result in L2 geminate productions by learners whose L1 exhibits a quantity distinction/length contrast. However, Mitterer (2021) found that exposure to orthographic input in English does not always result in such a length distinction. In words with a single grapheme vs. a digraph, Maltese-speaking L2 English learners produced target-like length despite their L1’s contrast.

Like Italian, Maltese has a quantity contrast; therefore, one would expect that there would be orthography-induced first language phonological transfer. The lack of an influence of orthography was attributed to early English acquisition in Malta, where spontaneous usage outside of the classroom occurs. The author argued that L2 orthographic effects are not automatic but may be related to the type of acquisition of the L2.

The effect of orthography on the production of an L1-induced length contrast in English has also been tested in L2 Levantine Arabic-speaking learners of English by Abu Guba (2023). Beginner, intermediate, and advanced learners were tested. Data were collected through a semistructured interview. The questions included target words that were expected to be realized with geminates such as “collect” and “select” by Levantine Arabic-speaking learners. The results showed that although orthography may potentially trigger geminate production, it alone does not explain the learners’ patterns of production. Specifically, the results showed that not all geminated words produced by the L2 learners are spelled with a digraph in English (e.g., “select”). Second, a large number of words written with a digraph (e.g., “sorry”) were produced with a singleton by the L2 learners. The author argued that the effect of orthography is constrained by the Arabic suprasegmental constraints. That is, the author put forth that gemination is primarily triggered by first language underrepresented structural rules as well as Universal Grammar (UG) marked-ness principles.

In addition to substitutions, there has been evidence of combined sound production. For example, Rafat and Stevenson (2018) further examined the data produced by participants in Rafat (2016) and found evidence of non-target-like combination productions in the production of the target sound /l/ that corresponds to the sound /j/ in Spanish and is represented with the digraph <ll> (e.g., */po**lj**o/ for <pollo> /po**j**o/). They attributed this type of production to a perceptual illusion, namely, a McGurk-like effect (McGurk & MacDonald, 1976).

In all, exposure to orthographic input can have a positive effect, can result in non-target-like productions, or have no effect. A number of studies in L2 production have indicated that exposure to orthographic input can trigger L1-phonological transfer, which can lead to sound additions, deletions, or substitutions. Some gaps in the literature remain. First, it has recently been suggested that exposure to orthographic input may result in a perceptual illusion which may lead to combination productions (McGurk & MacDonald, 1976). Second, most studies have examined orthographic effects in languages with Roman alphabets. The literature on the effect of L2 diacritics and different types of scripts is sparse. Therefore, the purpose of this study is to examine the effect of L2 diacritics on L2 geminate production in native speakers of two different languages (with different scripts): English (alphabetic) and Japanese (Kanji logographic and Kana syllabic) speakers learning Arabic (abjad).

## 6 Hypotheses

The following hypothesis regarding the effect of L1 phonetics and phonology on L2 geminate production in Arabic is tested in this study:

**H1.** According to the Revised Speech Learning Model (Flege & Bohn, 2021), sounds that exist in the L1 should not pose any difficulty for the learners in the L2. Therefore, Japanese L2 learners of Arabic (JLs) will be able to produce geminates in Arabic as an L2. However, English L2 learners (ELs) may not perceive the phonetic dissimilarity between the geminates and their nearest L1 sounds (singletons). Therefore, the ELs may not be able to produce Arabic geminates or may produce them differently from the native speakers of Arabic.

The hypothesis regarding the effect of orthographic input is as follows:

**H2.** Exposure to orthographic input will modulate Arabic geminate production by both JLs and ELs of Arabic (Escudero et al., 2008; Pattamadilok et al., 2021; Rafat, 2015; Showalter & Hayes-Harb, 2013; Steele, 2005). Specifically, the presence of the geminate diacritic will make the feature length more salient and therefore promote the production of geminates, but the absence of the geminate diacritic, when orthographic input is presented, will inhibit the accurate production of L2 geminates because the geminate-singleton contrast is neutralized in writing. The following hierarchy of difficulty is produced, where geminate production is the easiest from left to right:

Presence of orthographic input with the geminate diacritic > absence of orthographic input > presence of orthographic input without the geminate diacritic

## 7 Methodology

### 7.1 Participants

There were three groups of participants: (1) 15 ELs of Arabic (males = 6, females = 9), (2) 15 JLs of Arabic (males = 3, females = 12), and (3) 10 ACs (Arabic controls; males = 2, females = 8). They ranged in age from 18 to 44. Both the ELs and JLs were studying at the advanced level of Arabic and had spent approximately 2 to 5 years studying Arabic. The ELs were studying at the Arabic Flagship Program at Indiana University in the United States, whereas the JLs were studying at the Arabic Islamic Institute in Japan. For both the ELs and JLs, the age of onset of acquisition of Arabic was between 15 and 17 years old. The oldest participants had interrupted their learning of Arabic but, in the demographic survey, did not indicate for how long. The ACs were studying Arabic Studies at Princess Nourah bint Abdulrahman University in Saudi Arabia. They ranged in age from 20 to 30. Before enrollment, the ELs and JLs took a formal placement test conducted by the institutions. The placement test consisted of five sections: listening, reading, grammar, writing, and speaking. Both learner groups were placed at the advanced level. To obtain another measure of proficiency, based on Colantoni and Steele (2006) and Birdsong (2003), we had two native speakers of Arabic judge the learners’ and the control group’s native-like pronunciation. The judges rated the learners’ production of the reading task they had completed during the experiment (ortho-without-diacritics). The ACs were unanimously rated 5 of 5, whereas the ratings of the ELs (*M* = 4.10, *SD* = 0.80) and the JLs (*M* = 3.66, *SD* = 1.09) did not significantly differ, *t*_(28)_ = 1.2, *p* *=* .24) from each other. None of the participants reported having speech, language processing, or hearing disorders.

### 7.2 Stimuli

There were 24 Arabic target words in each task (12 singletons and 12 geminates), and each word was repeated 3 times, yielding 72 items per task per participant. The three tasks were (1) a delayed imitation task, (2) a reading task without diacritics, and (3) a reading task with diacritics. The target words were geminate-singleton minimal pairs (e.g., <قَدَمَ> /ˈ/ɑdɑmɑ/ “he came” vs. <قَدَّمَ> /ˈqɑdːɑmɑ/ “he gave”). That is, one word had the same phoneme and grapheme, but one word in the pair contained a singleton sound while the other contained a geminate sound. The target words were inserted in carrier sentences (for details, see the Supplementary Materials, Tables S2, S4, and S6).

The words were generated using four geminate-singleton stop consonants /b/-/bː/, /t/-/tː/, /d/-/dː/, and /k/-/kː/, a nasal stop consonant /m/-/mː/, and an emphatic stop consonant /tˤ/-/tˤː/ flanked by /ɑ/ on both sides (for details, see the Supplementary Materials, Tables S1, S3, and S5). The Arabic sound /tˤ/ is the only sound that is not part of the phonological systems of the ELs and JLs. The target stop sounds all occurred in the same intervocalic contexts (i.e., /ɑ/ C /ɑ/). Two pairs of words were included per target singleton and geminate sound in each task.

All target words in all three tasks were trisyllabic and represented a common environment in which geminates exist in Arabic. They had the same syllabic structure (CV.CV.CV for singletons and CVC.CV.CV for geminates, where V was the vowel /ɑ/), were stressed on the antepenultimate syllable (e.g., <قَدَمَ> /ˈqɑdɑmɑ/ “he came” vs. <قَدَّمَ>/ˈqɑdːɑmɑ/ “he gave”) and were in the simple past tense.

Two teachers from the institutions where the participants studied confirmed that the chosen target words were frequently used in their courses and speech, and that they were common simple words. They also stated that advanced learners would not have difficulty with the target words in the study, as all words occurred frequently in written and spoken Arabic. The only word used more frequently in written than in spoken language is /ʃɑmɑrɑ/ <شَمَرَ> “walked proudly,” which is used in the reading task diacritics (Task 2).

In the delayed imitation task, the target words were inserted in a carrier sentence. The following are examples of sentences containing one of the geminate-singleton pair words (for details, see the Supplementary Materials, Table S2):

1. <هَدَّفَ اللاَّعِب الكُرَة>

 /hɑ**dː**ɑfɑ ɑlɑʔɪb ɑlˈkʊrɑ/

   Geminate

 “The player caused the football to enter the goal line.”

2. <هَدَفَ الحِوَار إلَى السَّلَامِ>

 /hɑdɑfɑ alħiːˈwɑːr ɪlɑː ɑˈsːɑlɑmiː/

   Singleton

 “Dialogue is aimed to create peace.”

The target words were the same in both reading tasks: ortho-without-diacritics, and ortho-with-diacritics (for details, see the Supplementary Materials, Tables S3 and S5), but they were different from those in the delayed imitation task. This choice was made to avoid potential learning effects from the delayed imitation task. The target words differed between the ortho-without-diacritics and the ortho-with-diacritics tasks only in terms of writing. Gemination was not marked in the ortho-without-diacritics task (e.g., <سكر> /sɑkːɑrɑ/ “closed”) but was marked in the ortho-with-diacritics task (e.g., <سَكَّر>). In both reading tasks, the stimuli were inserted into carrier sentences (for details, see the Supplementary Materials, Tables S4 and S6, respectively). In the ortho-without-diacritics task, the words were inserted in carrier sentences that would provide the context for the target words since they were not marked with the geminate diacritic (e.g., <سكر الرجل الأبواب> /sɑkːɑrɑ arˈd͡ʒul al.ʔa'buːb/ “The man closed the doors”). In the ortho-with-diacritics task, they were inserted in the context-free carrier sentence <أنا أقول . . . مرة أخرى> /ɑnɑː ɑquːl . . . maˈratun uːx́rɑː/ “I say . . . once again” in Arabic.

The filler words were 12 real Arabic nonrhyming words (e.g., <يغني> /juːɣɑnːi/ “singing” and <لغات> /luːɣɑːt/ “languages”). The stimuli were recorded in Standard Arabic by a female native speaker of Najdi Arabic and were randomized.

### 7.3 Tasks

The procedures in the delayed imitation task, where learners were exposed to only auditory input, were adapted from Bassetti (2017). The purpose of this task was to examine the phonological representation of geminates across L2 learners. In the delayed imitation task, the participants were presented with a Microsoft PowerPoint (PPT). First, they listened 3 times to a sentence containing one of the geminate-singleton pair words through their headphones. Second, they heard the sentence with the missing target word in Arabic. Third, they were asked to count backward from 7 to 1 in Arabic and then repeat the target word in the sentence <. . . الكلمة الناقصة هي> /ɑlˈkɑliːmɑtuː ɑnɜːɑqiːsˤɑɦ ɦːˈæɑ/ “the missing word is . . .” three times. The goal of using backward counting was to remove traces of the phonological input from memory (Bassetti, 2017). The participants could hear the sentence again up to two more times by pressing the “play sound” button on the laptop screen.

In the ortho-without-diacritics task, where gemination was not indicated with diacritics, all participants were asked to read Arabic sentences with target words containing one of the geminate-singleton pair words without diacritics. The goal of this task was to determine whether exposure to orthographic input affects the production of Arabic geminates.

The ortho-with-diacritics task, where gemination was indicated with diacritics, was designed to test the effect of diacritics on the L2 production of Arabic geminates. This task was similar to the ortho-without-diacritics task. However, in this task, one of the geminate-singleton pair words was placed in the following carrier sentence in Arabic: <أنا أقول. . . مرة أخرى> /**ɑnɑː ɑquːl . . . mɑːrːɑtəʊn uːx́rɑ:/ “**I say . . . once again.”

In both reading tasks, the participants received three lists, with each list containing 36 sentences (12 geminate target words, 12 singleton target words, and 12 filler words). Each list was read 3 times. The sentence presentation order was randomized.

There were three repetitions in the three tasks, yielding 72 target tokens per participant, with the mean value of these three repetitions used for analysis. All participants were familiarized with each task prior to the experiment and were given short trials to practice the techniques. They also completed a background questionnaire eliciting information about their age, gender, and language experience and background. Prior to the three tasks, the participants were instructed to speak or read at a normal rate. They were recorded individually in a quiet room using an M-Audio Micro-track 24/96 professional two-channel mobile digital recorder and a lavaliere unidirectional microphone. The recordings were made at a sampling rate of 44.2 kHz and a quantization rate of 16 bits. The audio files containing the extracted tokens were downsampled at 22.1 kHz and saved in WAV format.

## 8 Data analysis

**Figure 1.**
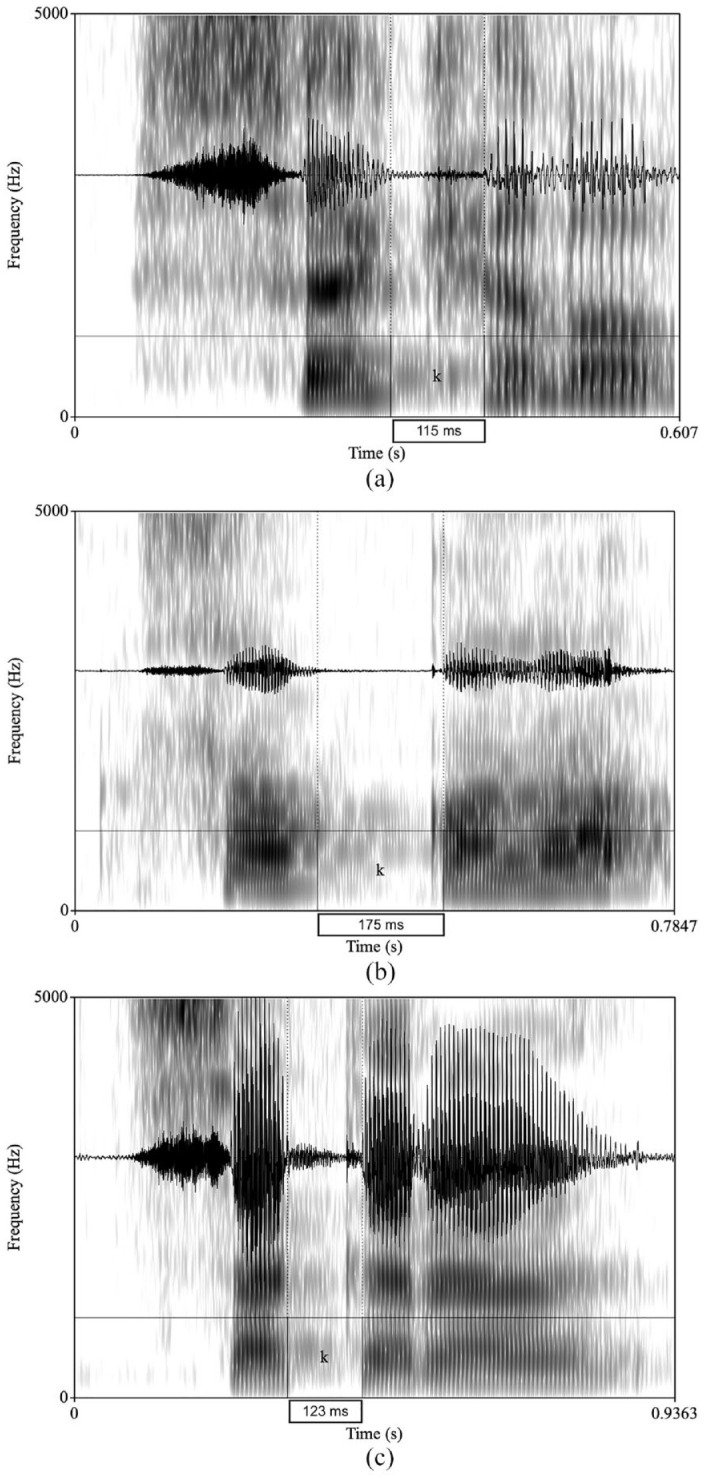
Examples of the singleton Arabic word <sa**k**ara > /sɑ**k**ɑrɑ/ produced by a participant from each speaker group. (a) Arabic control singleton sample. (b) Japanese learner singleton sample. (c) English learner singleton sample.

**Figure 2.**
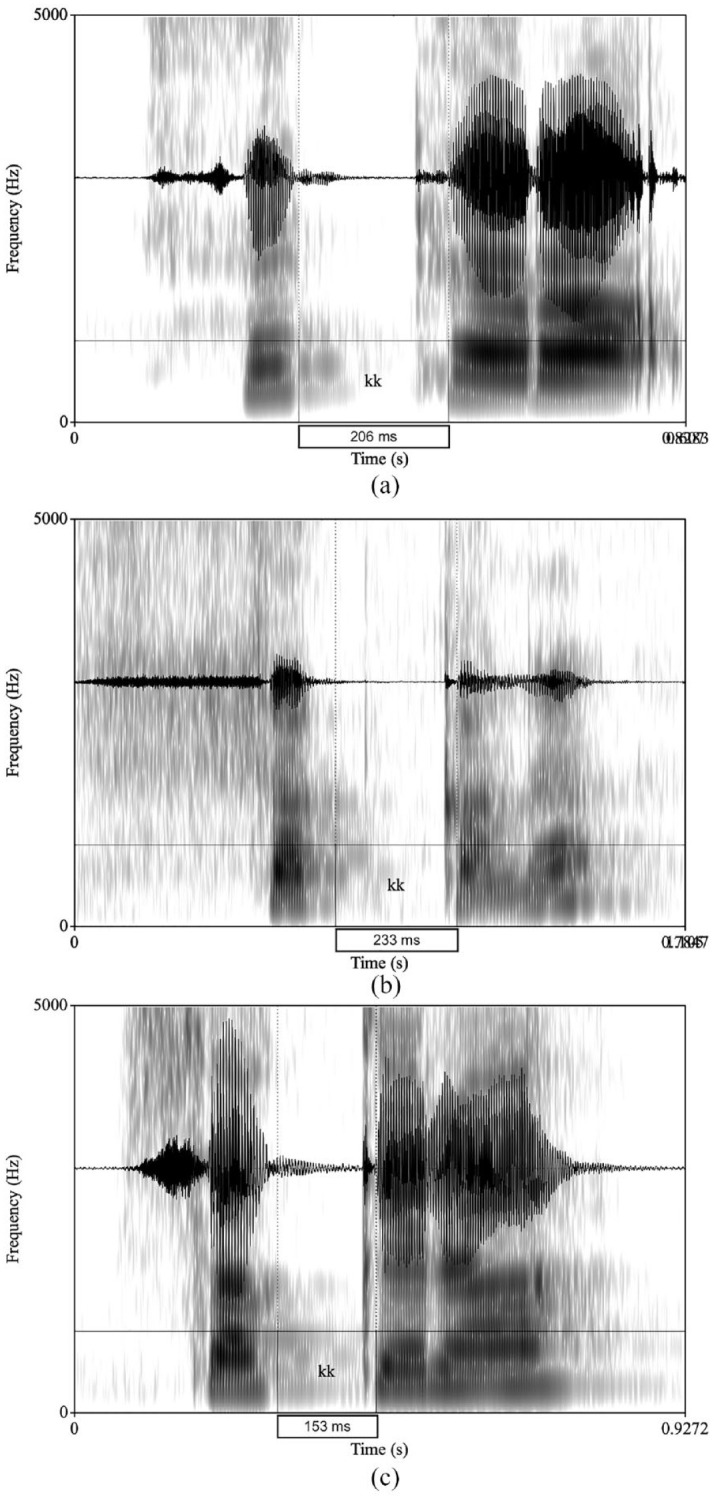
Example of the geminated word <sakkara > /sɑkːɑrɑ/. (a) Arabic control geminate sample. (b) Japanese learner geminate sample. (c) English learner geminate sample.

A total of 8,640 tokens were analyzed acoustically in PRAAT (Boersma & Weenink, 2012). The acoustic analysis of the target consonants was conducted by one of the authors, who is a native speaker of Arabic and an expert in acoustic analysis. We measured stop duration starting at the onset of the closure through aspiration and stopping at the periodic waveform (visible vowel). The geminate duration for /m/ was measured from the beginning to the end of the consonant. Figure 1 illustrates sample waveforms and spectrograms of the singleton Arabic word /sɑkɑrɑ/ <sakara>. Figure 2 illustrates sample waveforms and spectrograms of the geminate Arabic word /sɑkːɑrɑ/ <sakkara> .

Figure 1 depicts the onset and the offset of the singleton stop /k/ which are marked by the dotted lines, and the duration which is shown in the box in a token produced by a participant from (a) the ACs group (115 ms), (b) the JLs group (175 ms), and (c) the ELs group (123 ms).

Figure 2 depicts the onset and the offset of the geminate stop /kː/ which are marked by the dotted lines, and the duration which is shown in the box in a token produced by a participant from (a) the ACs group (206 ms), (b) the JLs group (233 ms), and (c) the ELs group (153 ms).

Target consonants were analyzed acoustically in the three tasks. The mean durations of singletons and geminates were calculated for all three groups. The AC group’s data were used as the standard for accurate production of singleton and geminate sounds. To address the research hypotheses in this study, multilevel modeling (MLM) analysis was employed. This analysis allows to account for a nested structure of the data (each person pronounced multiple words in the three tasks). A two-level multilevel model with persons at level 2 and words at level 1 was performed. Duration (ms) was used as a dependent variable, while group (AC, EL, and JL), task (1, 2, and 3), and type of sound (singleton vs. geminate) were used as independent variables. Two- and three-way interactions between the group and two other variables were also included in the model. In addition, we investigated geminate-singleton differences in duration. To examine these differences, one-way ANOVAs with group as a between factor were performed for each task.

Stata 17 was used to perform this analysis. Specifically, *xtmixed* command was used to run the MLM and *pwcompare* command was used to perform the pairwise comparisons for significant effects in the MLM model. In the following section, we report the results.

## 9 Results

The results of MLM analysis are presented in Table 1. As the table indicates both random effects are significant, indicating that both the participants and the multiple words they pronounced served as a source of variance in the data. However, the within-participants variance component is considerably larger than the between-participants variance component, accounting for 79.2% of variance in the data. The main effects of group (i.e., ACs, JLs, and ELs) and sound type (i.e., geminates and singletons) are significant. However, the three-way interaction effect, as well as both two-way interactions, are significant as well, preventing valid interpretation of the main effects.

**Table 1.** The Results of MLM Analysis.

Predictor variable	Estimate	*SE*	z	*p*	95% CI	
Intercept	107.54	8.73	12.33	.000	90.44	124.65
Group (reference group—ACs)						
(EL)	29.60	11.29	2.62	.009	7.48	51.73
(JL)	52.12	11.44	4.56	.000	29.70	74.53
Task (reference category—task 1 “Imitate”)						
Task 2 “No-Ortho”	9.16	6.04	1.51	.13	−2.69	21.00
Task 3 “Ortho”	14.45	6.06	2.39	.017	2.58	26.32
Sound Type(reference category—singleton)						
Geminate	99.03	6.06	16.35	.000	87.16	110.90
Group × Task						
EL × Task 2 “No-Ortho”	−29.43	7.84	−3.75	.000	−44.80	−14.06
EL × Task 3 “Ortho”	−37.43	7.85	−4.77	.000	−52.82	−22.04
JL × Task 2 “No-Ortho”	14.67	8.06	1.82	.069	−1.12	30.47
JL × Task 3 “Ortho”	4.44	8.06	0.55	.582	−11.36	20.25
Group × Sound Type						
EL × Geminate	−70.73	7.88	−8.98	.000	−86.17	−55.29
JL × Geminate	−14.63	8.32	−1.76	.079	−30.94	1.68
Task × Sound Type						
Task 2 “No-Ortho” × Geminate	−10.90	8.56	−1.27	.203	−27.67	5.87
Task 3 “Ortho” × Geminate	−14.00	8.57	−1.63	.102	−30.79	2.79
Group × Task × Sound Type						
EL × 2 “No-Ortho” × Geminate	1.08	11.09	0.10	.922	−20.66	22.82
EL × 3 “Ortho” × Geminate	25.24	11.10	2.27	.023	3.48	46.99
JL × 2 “No-Ortho” × Geminate	−31.78	11.42	−2.78	.005	−54.17	−9.39
JL × 3 “Ortho” × Geminate	−5.73	11.42	−0.50	.616	−28.12	16.65
Random effects						
Variance between participants	577.89	136.72	4.23	.000	363.46	918.82
Variance within participants	2,201.13	59.51	36.99	.000	2,087.52	2,320.92

**Figure 3.**
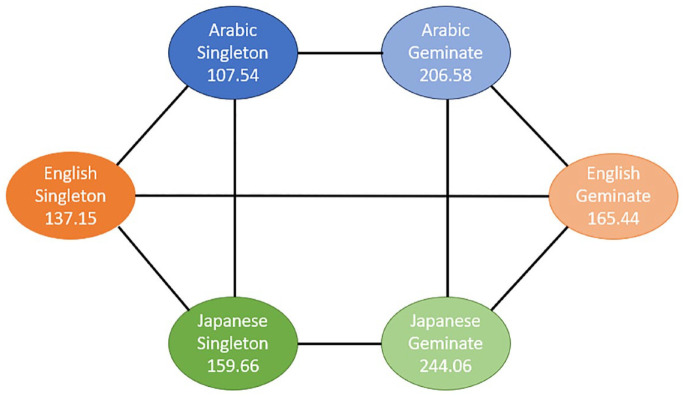
Pairwise comparisons of singleton and geminate sounds within and across the three study groups for Task 1 “Imitate.”

**Figure 4.**
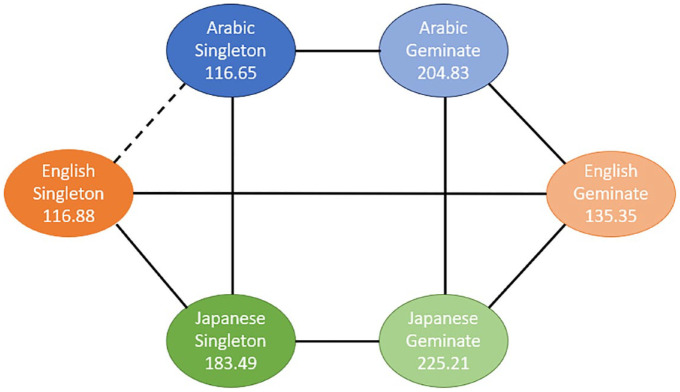
Pairwise comparisons of singleton and geminate sounds within and across the three study groups for Task 2 “No-Ortho.”

**Figure 5.**
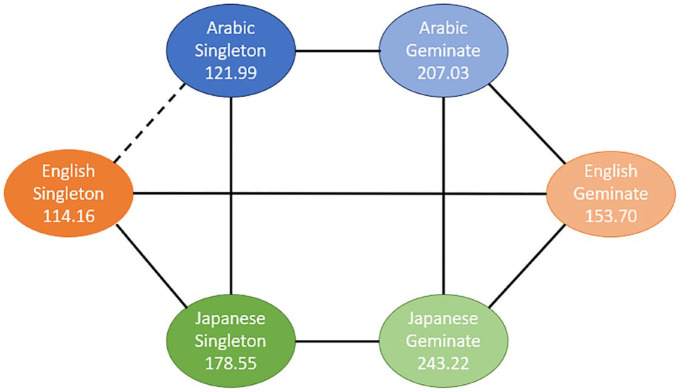
Pairwise comparisons of singleton and geminate sounds within and across the three study groups for Task 3 “Ortho.”

**Figure 6.**
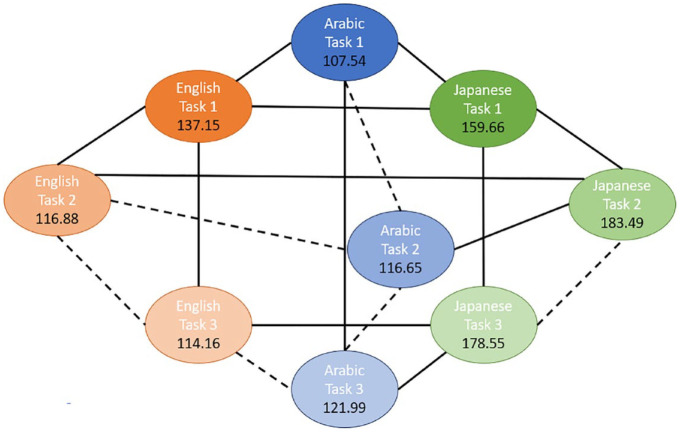
Pairwise comparisons of the three tasks for singleton sounds within and across the three study groups.

**Figure 7.**
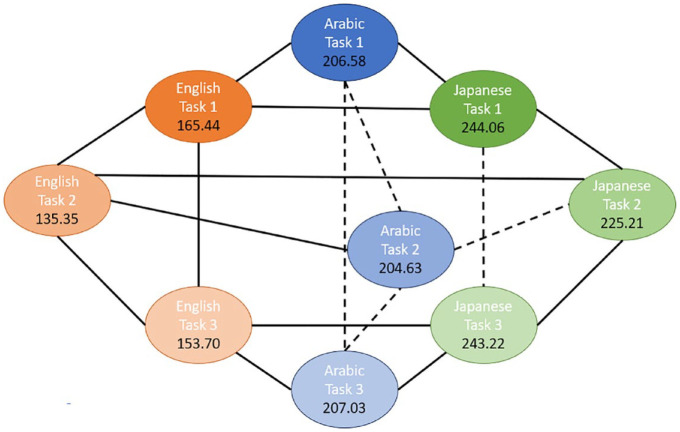
Pairwise comparisons of the three tasks for geminate sounds within and across the three study groups.

To further investigate these significant interactions, pairwise comparisons were conducted. Because the higher-order (three-way) interaction was significant, the interpretation of the results is based on the pairwise comparisons for this interaction. To ease the interpretation, we present the results for two different data dimensions: (1) comparison of singleton and geminate sounds across the three groups for each task (Figures 3 to 5) and (2) comparison of the three tasks across the study groups for each sound type (Figures 6 and 7). The mean sound duration (in ms) is displayed for each comparison group. The comparison groups that are significantly different from each other are connected with a solid line, whereas the comparison groups that are not significantly different from each other are connected with a dashed line. Different colors are used to depict the three groups (blue for AC, orange for EL, and green for JL), and different shades of the same color are used to depict the types of sound (darker for singleton and lighter for geminate) and the three tasks.

As can be seen from Figures 3 to 5, there is a significant difference between the duration of singleton and geminate sounds in all three study groups for all three tasks. Specifically, the average duration of geminates is longer than the average duration of singletons: the light circles are connected by a solid line to their dark counterpart. In the comparisons between groups in each task, the EL group has a significantly shorter average duration for geminate sounds compared with the AC group. Conversely, the Japanese learners, on average, have a longer duration of geminates compared to the AC group on all three tasks. In addition, the average duration of singleton sounds is significantly longer for the JL group compared with the ACs and ELs on all three tasks. However, while the ELs have significantly longer duration of singletons in the imitation task (Figure 3), in the reading tasks, the difference between the EL and AC groups on this type of sound is nonsignificant (Figures 4 and 5).

As can be seen from Figures 4 and 5, the differences between the three study conditions vary among the groups of EL, JL, and AC groups for singleton and geminate sounds.

### 9.1 Singleton sounds

ELs produced significantly longer sounds in Task 1 “a delayed imitation task” comparing to both Tasks 2 “a reading task without diacritics” and 3 “a reading task with diacritics.” However, the sound duration is not significantly different between Tasks 2 and 3 in this group. In contrast, in the AC group, sound duration for Task 1 was significantly shorter than in Task 3, whereas it was not significantly different from the average sound duration in Task 2. There was no significant difference in sound duration between Tasks 2 and 3 in this group as well. Finally, JLs produced significantly shorter sounds in Task 1 compared to both Tasks 2 and 3, but there was no significant difference between these tasks.

In Task 1, JLs produced the longest singleton sounds, followed by the ELs. The ACs produced the shortest singletons in this task. JLs also produced significantly longer singleton sounds in Task 2 compared with both other study groups. However, the difference in sound duration was not significant between the EL and AC groups for this task. Similar results are observed for Task 3.

### 9.2 Geminate sounds

ELs produced significantly longer geminate sounds in Task 1 compared with the reading tasks. They also produced significantly longer sounds in Task 3 compared with Task 2. In contrast, in the AC group, the average geminate duration was not significantly different across the three tasks. Finally, JLs produced significantly shorter geminate sounds in Task 2 compared with both Tasks 1 and 3, but there was no significant difference between these tasks.

In Task 1, JLs produced the longest geminates, followed by the Arabic Learners. The ELs produced the shortest geminates in this task. Similar results are observed for Task 3. However, ELs produced significantly shorter geminate sounds in Task 2 compared with both other study groups. The difference in sound duration was not significant between the JL and AC groups for this task.

### 9.3 Geminate-singleton differences

One potential explanation of significant 3-way interaction between the type of speaker (Arabic, Japanese, and English), type of sound (geminate or singleton), and task (1, 2, and 3) is the difference in the magnitude of the singleton-geminate difference between the three types of speakers in the three tasks. To investigate this hypothesis, the average sound duration was computed for each participant for the two types of sounds and the three tasks. Then the duration difference scores between geminate and singleton sounds were computed for the three tasks for each participant. The descriptive statistics for the difference scores are displayed in Table 2. As can be seen from this table, the pattern of differences in the duration of the geminate-singleton difference scores among the three groups vary by task.

**Table 2.** Difference Scores in Duration of Geminates and Singletons for Each Task for Arabic, English, and Japanese speakers.

Task	Group	N	Mean	*SD*	*SE*	Min	Max
Task 1	Arabic	10	0.10	0.02	0.01	0.07	0.12
	Japanese	15	0.08	0.04	0.01	0.02	0.15
	English	15	0.03	0.04	0.01	−0.05	0.11
Task 2	Arabic	10	0.09	0.01	0.00	0.06	0.11
	Japanese	15	0.04	0.05	0.01	−0.02	0.13
	English	15	0.02	0.04	0.01	−0.01	0.11
Task 3	Arabic	10	0.09	0.02	0.01	0.05	0.10
	Japanese	15	0.06	0.03	0.01	0.01	0.11
	English	15	0.04	0.03	0.01	0.00	0.11

To further investigate these differences, one-way ANOVAs with group as a between factor were performed for each task. The assumption of equal variance as measured by Levene’s tests was not violated for any of the ANOVAs; therefore, Bonferroni post hoc tests were performed. The results of all three ANOVAs were significant (Table 3).

**Table 3.** ANOVA Tests Comparing the Average Geminate-Singleton Differences Between the Three Groups.

Task	Variance decomposition	SS	df	MS	F	*P* value
Task 1	Between Groups	0.035	2	0.017	13.939	<.001
	Within Groups	0.046	37	0.001		
	Total	0.081	39			
Task 2	Between Groups	0.030	2	0.015	10.241	<.001
	Within Groups	0.054	37	0.001		
	Total	0.084	39			
Task 3	Between Groups	0.013	2	0.006	7.444	.002
	Within Groups	0.032	37	0.001		
	Total	0.045	39			

**Figure 8.**
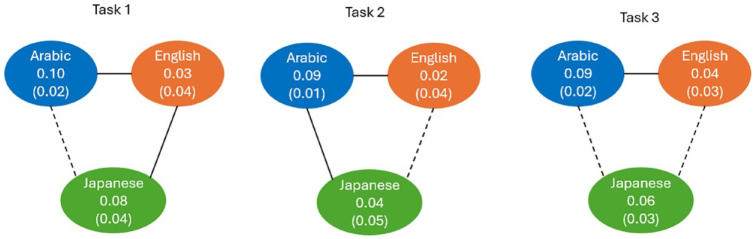
Pairwise comparisons of the mean differences in geminates-singleton duration between Arabic, English, and Japanese groups of speakers.

The results of post hoc comparisons using Bonferroni method are displayed in Figure 8. Solid lines represent significant differences, whereas dashed lines represent nonsignificant differences.

As can be seen from this Figure, for Task 1, English speakers had a significantly smaller difference in geminates-singleton duration comparing to both other groups. In contrast, for Task 2, Arabic speakers had a significantly larger difference in geminates-singleton duration compared with the other two groups. Finally, for Task 3, English speakers had significantly smaller difference in geminates-singleton duration compared with Arabic speakers only.

## 10 Discussion

This study posed two research questions: (1) Is there an effect of L1 phonology on the L2 production of Arabic geminate consonants? This study investigated whether L2 Arabic geminate consonant production was difficult for both JLs and Els. (2) Is there an effect of exposure to orthographic input? Specifically, is there a difference between Arabic geminate consonant production under different conditions, such as when L2 learners imitate Arabic geminates without exposure to orthographic input, read Arabic geminates without diacritics, or read Arabic geminates with diacritics?

These research questions are addressed here. Based on the SLM-*r* (Flege & Bohn, 2021), we predicted that JLs would be able to produce L2 Arabic geminates but ELs would either not be able to produce them or would produce them differently in comparison to the ACs. It was also predicted that exposure to orthographic input would modulate Arabic geminate production by both JLs and ELs of Arabic (Escudero et al., 2008; Pattamadilok et al., 2021; Rafat, 2015; Showalter & Hayes-Harb, 2013; Steele, 2005). Specifically, it was predicted that the presence of the geminate diacritic would make the feature length more salient and therefore promote the production of geminates. However, the absence of the geminate diacritic, when orthographic input is presented, would inhibit the accurate production of L2 geminates because the geminate-singleton contrast is neutralized in writing. These hypotheses were partially confirmed. The results of the geminate-singleton differences analysis showed that both learner groups were able to produce geminates; however, the JLs exhibited an advantage over the ELs in Task 1 and seemed to benefit more than the ELs from the presence of diacritics in Task 3 (ortho with diacritics).

These findings are important because they highlight the role of L1 phonology in L2 speech learning. Particularly, these results show that the existence of the geminate-singleton contrast in the L1 aids with the production of geminates in a new language (i.e., Arabic), even if the contrast is phonetically realized differently. The fact that L2 geminates may differ in length in comparison with the geminates of the target language has been shown in previous studies. Similar to the findings in the current study, geminates have also been shown to be both hyper- and hypoarticulated. For example, Spanish and German learners of Italian (Cordero et al., 2018; Sorianello, 2014) have been reported to hypoarticulate the Italian geminates. Similarly, intermediate English learners of Japanese have been shown to hypoarticulate Japanese geminate consonants (M. S. Han, 1992), but an advanced learner has been shown to hyperarticulate them (Mah & Archibald, 2003). In addition, Colombian Spanish speakers both hypo- and hyperarticulated the Italian and Cuban Spanish geminates (Cordero et al., 2018).

Nonetheless, it is important to note that, in this study, we assumed that geminates do not exist word internally in the intervocalic position in English; however, this issue needs further examination in future studies, as it is our intuition that geminates may sometimes occur in child-directed and emphatic speech, particularly in women’s speech.

With respect to the effect of orthography, our results indicated language-specific task effects. Specifically, as mentioned above, the JLs benefited more than the ELs from the presence of diacritics. Previously, other studies have also reported an effect of orthography on more advanced learners (e.g., Bassetti, 2017; Bassetti & Atkinson, 2015; Rafat et al., 2021; Sokolović-Perović et al., 2020). However, previous studies have looked at the effect of orthography in the ELs and JLs separately. For example, Showalter and Hayes-Harb (2013) showed that beginner ELs used diacritics when exposed to Chinese using the same Latinized alphabetic system. Moreover, Sokolović-Perović et al. (2020) had reported that highly proficient Japanese learners of English were affected by English orthography. However, they examined the effect of digraphs, and not diacritics. In addition, to the best of our knowledge, this is the first study that has compared JLs with ELs with respect to the effect of diacritics in a language with a different alphabetic system (i.e., an abjad).

Regarding the ACs, as expected, they were consistently able to produce geminates across the three tasks and the differences between their geminate singleton productions did not significantly differ between the three tasks.

Notably, in this study, we compared the results of a delayed imitation task with those of two reading tasks, where learners did not hear the target words and the tasks also involved different target items and carrier sentences. To make the results more comparable, future studies can replicate the study by including an auditory-only delayed imitation task and two auditory-orthographic tasks, one where geminates are represented with a diacritic and another without the diacritic. Future studies could also control carrier sentences by including the same sentences in all tasks. Finally, non-target-like productions of the geminates in this study did not seem to affect the raters’ assessment of their level of proficiency. Future research should investigate whether the production of geminates influences perceived accentedness.

## 11 Conclusion

This study compared Arabic geminate stop consonant production by two different learner groups with typologically different languages, namely, English and Japanese and found language-specific effects. The overall findings confirm that both learner groups were able to produce geminates, although the JLs exhibited an advantage over the ELs in the auditory-only task and in the presence of the diacritics. Moreover, the phonetic realizations of both groups were different from those of the ACs.

The current research is one of the few studies to have examined L2 geminate production by learners of typologically distinct languages and to have shown orthographic effects with respect to diacritics in a cross-script context (i.e., English and Arabic). Most previous studies have focused on languages with a Roman alphabet. More studies on the effect of orthographic input on L2 speech learning are needed to further illuminate the undeniably complex relationship between different L1 and L2 orthographic systems and the effect of orthographic input on L2 production. Research is especially lacking regarding diacritics, types of scripts, and the level of proficiency of the learners. The role of orthography also needs to be addressed in future L2 models of acquisition.

## Supplemental Material

sj-docx-1-las-10.1177_00238309241267876 – Supplemental material for An Investigation of Language-Specific and Orthographic Effects in L2 Arabic geminate production by Advanced Japanese- and English-speaking learners Supplemental material, sj-docx-1-las-10.1177_00238309241267876 for An Investigation of Language-Specific and Orthographic Effects in L2 Arabic geminate production by Advanced Japanese- and English-speaking learners by Albandary Aldossari, Ryan Andrew Stevenson and Yasaman Rafat in Language and Speech
