# Supplementary material for: An Investigation of Language-Specific and Orthographic Effects in L2 Arabic geminate production by Advanced Japanese- and English-speaking learners
Source: Lang Speech. 2024 Aug 30;68(2):365–90. doi: 10.1177/00238309241267876 (PMC12106925; doi:10.1177/00238309241267876)
Supplement: sj-docx-1-las-10.1177_00238309241267876 – Supplemental material for An Investigation of Language-Specific and Orthographic Effects in L2 Arabic geminate production by Advanced Japanese- and English-speaking learners [file sj-docx-1-las-10.1177_00238309241267876.docx]

**Appendices**

**Supplement S1**

| Table S1. | | *Task (1) a delayed imitation task: target words* | |
| --- | --- | --- | --- |
|  | | **Mid Singleton Words** | **Mid Geminate Words** |
| **/d/** | **1** | /hɑ**d**ɑfɑ/ <هَدَفَ> “aimed” | /hɑ**dː**ɑfɑ/ <هَدَّفَ> “entered” “the football” |
|  | **2** | /ʔɑ**d**ɑlɑ/ عَدَلَ>> “changed” | /ʔɑ**dː**ɑlɑ/ عَدَّلَ> > “modified” |
| **/b/** | **3** | /sˤɑ**b**ɑrɑ/ <صَبَرَ> “was patient” | /sˤɑ**bː**ɑrɑ/ صَبَّرَ> > “consoled” |
|  | **4** | /θɑ**b**ɑtɑ/ <ثَبَتَ> “became strong” | /θɑ**bː**ɑtɑ/ <ثَبَّتَ> “fixed” |
| **/m/** | **5** | /sɑ**m**ɑʔɑ/ سَمَعَ> > “heard” | /sɑ**mː**ɑʔɑ/ <سَمَّعَ> “caused someone to listen to something” |
|  | **6** | /ħɑ**m**ɑlɑ/ حَمَلَ> > “carried” | /ħɑ**mː**ɑlɑ/ <حَمَّلَ> “imposed” |
| **/k/** | **7** | /ħɑ**k**ɑmɑ/ حَكَمَ> > “judged” | /ħ**ɑkː**ɑmɑ/ <حَكَّمَ> “refereed” |
|  | **8** | /ðɑ**k**ɑrɑ/ <ذَكَرَ> “mentioned” | /ðɑ**kː**ɑrɑ/ <ذَكَّرَ> “someone reminded someone else of something” |
| **/t/** | **9** | /wɑ**t**ɑrɑ/ <وَتَرَ> “prayed one prayer” | /w**ɑtː**ɑrɑ/ <وَتَّرَ> “conflicted” |
|  | **10** | /kɑ**t**ɑbɑ/ <كَتَبَ> “wrote” | kɑ**tː**ɑbɑ/ <كَتَّبَ> “someone asked someone else to write” |
| **/tˁ/** | **11** | /ʔɑ**tˤ**ɑlɑ/ <عَطَلَ> “broke down” | /ʔɑ**tˤː**ɑlɑ/ <عَطَّلَ> “delayed” |
|  | **12** | /ɣɑ**tˤ**ɑsɑ / <غَطَسَ> “dived” | /ɣɑ**tˤː**ɑsɑ / <غَطَّسَ> “someone caused someone else to drown” |

**Supplement S2**

| Table S2. | *Task (1) a delayed imitation task: carrier sentences* | | |
| --- | --- | --- | --- |
| **Read the following sentences:** | | | |
|  | **Transcription** | **Arabic sentences** | **Meaning** |
| **1** | /**hɑdɑfɑ** alħiːˈwɑːr ɪlɑː ɑˈsːɑlɑmiː/ | **هدف** الحوار إلى السلام. | Dialogue was **aimed to** create peace. |
| **2** | /**sɑmːɑʔɑ** arˈd͡ʒul sˁɑdɪqʊh/ | **سمع** الرجل صديقه. | The man **caused his** friend to **listen**. |
| **3** | /**ħɑkːɑmɑ** ɑlmʊdɑqɪq ɑlkɪtɑb/ | **حكم** المدقق الكتاب. | The editor **refereed** the book. |
| **4** | /**ðɑkɑrɑ** ɑlmuː'mɪn ɑllɑh/ | **ذكر** المؤمن الله. | The believer **mentioned** God. |
| **5** | /**ħɑkɑmɑ** ɑlqɑḍɪ bɪ' ɑlʔɑdl/ | **حكم** القاضي بالعدل. | The lawyer **judged with** justice. |
| **6** | /**ɣɑtˤːɑsɑ** ɑ'sɑbɑh sˁɑdɪqʊh/ | **غطس** السباح صديقه. | The swimmer **caused** his friend **to drown.** |
| **7** | /**θɑbːɑtɑ** ɑ'nɑgɑːrʊ ɑlmɪːsmɑrɑ/ | **ثبت** النجار المسمار. | The carpenter **fixed** the pin. |
| **8** | /**wɑtɑr**arˈd͡ʒul sˁɑlɑtʊh/ | **وتر** الرجل صلاته. | The man **prayed one prayer**. |
| **9** | /**kɑtɑbɑ** ɑlwɑlɑdʊ wɑgɪːbʊh/ | **كتب** الولد واجبه. | The boy **wrote** his assignment. |
| **10** | /**sˤɑbːɑrɑ** ɑ'sˤɑdiːq sˁɑdɪqʊh/ | **صبر** الصديق صديقه. | The friend **consoled** his friend. |
| **11** | /**ɣɑtˤɑsɑ** arˈd͡ʒul fɪː ɑlbɑhri/ | **غطس** الرجل في البحر. | The man **dived** into the sea. |
| **12** | /**ʔɑdːɑlɑ** ɑlmɔhɑndis ɑlbɪnɑ'/ | **عدل** المهندس البناء. | The engineer **modified** the construction. |
| **13** | **/ħɑmɑlɑ** ɑˈrɑdʒuːluː ɑlˈhɑqiːbɑh/ | **حمل** الرجل الحقيبة. | The man **carried** the bag. |
| **14** | /**ʔɑtˤːɑlɑ** ɑ'tˤːɑɪːrʊ ɑlrɪhlɑ/ | **عطل** الطيار الرحلة. | The pilot **disrupted** the flight. |
| **15** | /**ʔɑdɑlɑ** ɑ'sɑiːqʊ ʔɑn ɑltɑriqi/ | **عدل** السائق عن الطريق. | The driver **changed** his way. |
| **16** | /**ðɑkːɑrɑ** ɑlḵāʾtɪːbʊ ɑ'nɑs/ | **ذكر** الخطيب الناس. | The speaker **reminded** people (of something). |
| **17** | /**θɑbɑtɑ** ɑlmuː'mɪn ʔiːndɑ ɑlmʊsiːbɑ/ | **ثبت** المؤمن عند المصيبة. | The believer **became strong** when the calamity happened. |
| **18** | /**ʔɑtˤɑlɑ** ɑlgiːhɑz ɑlɪːum/ | **عطل** الجهاز اليوم. | The device **broke down** today. |
| **19** | /**sˤɑbɑrɑ** arˈd͡ʒul kɑθɪːrɑ/ | **صبر** الرجل كثيراً. | The man **was** so **patient**. |
| **20** | /**ħɑmːɑlɑ** ʔɑliːnɑ ɑlmɑhɑm/ | **حمل** علينا المهام. | He **imposed** us with tasks. |
| **21** | /**sɑmɑʔɑ** alwɑlɑdʊ ɑlmiːðiɑʔ/ | **سمع** الولد المذياع. | The boy **heard** the radio. |
| **22** | /**kɑtːɑbɑ** ɑlmʊʔɑliːm ɑ'tɑlɑmiːð/ | **كتب** المعلم التلاميذ. | The teacher **asked** students **to write**. |
| **23** | **/hɑdːɑfɑ** ɑlɑʔɪb ɑlˈkʊrɑ/ | **هدف** اللاعب الكرة. | The player **caused the** football **to enter** the goal. |
| **24** | /**wɑtːɑrɑ** ɑlʔɑdɔː ɑlʔiːlɑqɑt/ | **وتر** العدو العلاقات. | The enemy **conflicted** the relationships. |

**Supplement S3**

| Table S3. | | *Stimuli: Task (2) a reading task “ortho-without-diacritics”: target words* | |
| --- | --- | --- | --- |
|  | | **Mid Singleton Words** | **Mid Geminate Words** |
| **/d/** | **1** | /ħɑ**d**ɑθɑ/ حدث>> “happened” | /ħɑ**dː**ɑθɑ/ <حدث> “told” |
|  | **2** | /qɑ**d**ɑrɑ/ <قدر> “was able” | /qɑ**dː**ɑrɑ/ قدر>> “estimated” |
| **/b/** | **3** | /ʔɑ**b**ɑdɑ عبد>> “worshiped” | /ʔɑ**bː**ɑdɑ/ <عبد> “built” |
|  | **4** | /lɑ**b**ɑsɑ/ <لبس> “put on” | /lɑ**bː**ɑsɑ/ لبس>> “put some clothes on someone” |
| **/m/** | **5** | /sɑ**m**ɑrɑ/ <سمر> “spent his night time” | /sɑ**mː**ɑrɑ/ <سمر> “burned” |
|  | **6** | /ʃɑ**m**ɑrɑ/ <شمر> “walked proudly” | /ʃɑ**mː**ɑrɑ/ <شمر> “rolled something up” |
| **/k/** | **7** | /sɑ**k**ɑrɑ/ سكر>> “got drunk” | /sɑ**kː**ɑrɑ/ سكر>> “closed” |
|  | **8** | /rɑ**k**ɑzɑ/ ركز>> “established” | /rɑ**kː**ɑzɑ/ <ركز> “concentrated” |
| **/t/** | **9** | /kɑ**t**ɑmɑ/ <كتم> “kept” | /kɑ**tː**ɑmɑ/ كتم>> “prevented” |
|  | **10** | /ʔɑ**t**ɑbɑ/ عتب>> “blamed” | /ʔɑ**tː**ɑbɑ/ عتب>> “built a doorstep” |
| **/tˁ/** | **11** | /fɑ**tˤ**ɑrɑ/ فطر>> “sorrowed” | /fɑ**tˤː**ɑrɑ/ <فطر> “fed” |
|  | **12** | /nɑ**tˤ**ɑkɑ/ نطق>> “pronounced” | /nɑ**tˤː**ɑkɑ/ نطق>> “someone made someone else to utter” |

**Supplement S4**

| Table S4. | | *Task (2) a reading task “ortho-without-diacritics”: carrier sentences* | | |
| --- | --- | --- | --- | --- |
| **Read the following sentences:** | | | | |
|  | **Transcription** | | **Arabic sentences** | **Meaning** |
| **1** | /**lɑbɑsɑ** ɑ'tˤiːfl qɑmisʊh/ | | **لبس** الطفل قميصه. | The child **put** his shirt **on.** |
| **2** | **/fɑtˤːɑrɑ** mohammed ɑ'sɑiːm/ | | **فطر** محمد الصائم | Mohammed **fed** the fasting Muslim. |
| **3** | **/ʃɑmɑrɑ** ɑlq'ɪd/ | | **شمر** القائد. | The leader **walked proudly**. |
| **4** | /**lɑbːɑsɑ** ɑlɑbʊ ɪːbnɑh mɪ̈dɑliɑ/ | | **لبس** الأب ابنه ميدالية. | The father **put** a medal **on** his son. |
| **5** | **/ʃɑmːɑrɑ** ɑ'θɑɔ̃b/ | | **شمر** الثوب. | Someone **rolled** his clothes **up**. |
| **6** | /**rɑkɑzɑ** ɑ'tlmɪːð ʔɑlɑ a'ʃɑrh/ | | **ركز** التلميذ على الشرح. | The student **concentrated on** the explanation. |
| **7** | /**ʔɑtɑbɑ** mohammed ʔɑlɑ khalid/ | | **عتب** محمد على خالد. | Mohammed **blamed** Khalid. |
| **8** | /**ħɑdɑθɑ** tɑɣɑiːɔ̃r fiː ɑlbilɑd/ | | **حدث** تغير في البلاد. | A change **happened** in the country. |
| **9** | /**nɑtˤːɑkɑ** ɑlmʊdɑris ɑ'tˤfl/ | | **نطق** المدرس الطفل. | The teacher **made** the child **to utter**. |
| **10** | /**ʔɑbɑdɑ** ɑlmɑḵālɔ̃q ɑlḵāliq/ | | **عبد** المخلوق الخالق. | The creature **worshiped** the creator. |
| **11** | /**ħɑdːɑθɑ** mohammed khalid ʔɑn mɑʃrɔ̃ʔh ɑlgɑdiːd/ | | **حدث** محمد خالد عن مشروعه الجديد. | Mohammed **told** Khalid about his new project. |
| **12** | /**fɑtˤɑrɑ** ɑlhɔzn qɑlbʊh/ | | **فطر** الحزن قلبه. | The sadness **sorrowed** his heart. |
| **13** | /**ʔɑtːɑbɑ**arˈd͡ʒul ɑlbɑb ɑlḵāɑriːgɪ/ | | **عتب** الرجل الباب الخارجي. | The man **built a doorstep** at the external door. |
| **14** | /**sɑkːɑrɑ** arˈd͡ʒul al.ʔa'buːb/ | | **سكر** الرجل الأبواب. | The man **closed** the doors. |
| **15** | /**sɑmːɑrɑ** ɑlḵāɑbɑz ɑlḵāɔbz/ | | **سمر** الخباز الخبز. | The baker **burned** the bread. |
| **16** | /**kɑtɑmɑ** arˈd͡ʒul sɪːrɑh/ | | **كتم** الرجل سره. | The man **kept** his secret. |
| **17** | /**qɑdɑrɑ** ɑltɑliːb ʔɑlɑ ɪqnɑʔ zɑmɪlɔh/ | | **قدر** الطالب على إقناع زميله. | The student **was able** to convince his friend. |
| **18** | /**nɑtˤɑkɑ** ɑlmʊtɑʔɑliːm hɔrɔf gɑdɪːdɑh/ | | **نط**ق المتعلم حروف جديدة. | The learner **pronounced** new letters. |
| **19** | /**rɑkɑzɑ** arˈd͡ʒul ɑ'riːmh fiː ɑlɑrḍ/ | | **ركز** الرجل الرمح في الأرض. | The man **established** the spear in the ground. |
| **20** | /**qɑdːɑrɑ** ɑlmuʃriːf ɑlɔɑqt/ | | **قدر** المشرف الوقت. | The supervisor **estimated** the time. |
| **21** | /**ʔɑbːɑdɑ** ɑlmɑsɔ̃l tˤɔroqɑt ɑlmɑdinɑh/ | | **عبد** المسؤول طرقات المدينة. | The director **built** the city roads. |
| **22** | /**kɑtːɑmɑ** ɑl’iʔlɑmiː sˁɑdɪqʊh ʔn iːfʃɑ' ɑlḵɑbɑr/ | | **كتم** الإعلامي صديقه عن إفشاء الخبر. | The media-man **prevented** his friend to disclose the news. |
| **23** | /**sɑmɑrɑ** ɑlʃɑḵɑs mɑ'ʔ gɑlisɔh lɑilɑn/ | | **سمر** الشخص مع جليسه ليلاً. | The person **spent his night** with his friend. |
| **24** | /**sɑkɑrɑ**arˈd͡ʒul sɑbɑhɑn/ | | **سكر** الرجل صباحاً. | The man **got drunk** early in the morning. |

**Supplement S5**

| Table S5. | | *Stimuli: Task (3) a reading task “ortho-with-diacritics”: target words* | |
| --- | --- | --- | --- |
|  |  | **Mid Singleton Words** | **Mid Geminate Words** |
| **/d/** | **1** | /**ħɑdɑθɑ**/ <حَدَثَ> “happened” | /**ħɑdːɑθɑ**/ <حَدَّثَ> “told” |
|  | **2** | /**qɑdɑrɑ**/ <قَدَر> “was able” | /**qɑdːɑrɑ**/ <قَدَّرَ> “estimated” |
| **/b/** | **3** | /ʔ**ɑbɑdɑ**/ <عَبَدَ> “worshiped” | /ʔ**ɑbːɑdɑ**/ <عَبَّدَ> “built” |
|  | **4** | /**lɑbɑsɑ**/ <لَبَسَ> “put on” | /**lɑbːɑsɑ**/ <لَبَّسَ> “put some clothes on someone” |
| **/m/** | **5** | /**sɑmɑrɑ**/ <سَمَرَ> “spent his night time” | /**sɑmːɑrɑ**/ <سَمَّرَ> “burned” |
|  | **6** | /**ʃɑmɑrɑ**/ <شَمَرَ> “walked proudly” | /**ʃɑmːɑrɑ**/ <شَمَّرَ> “rolled something up” |
| **/k/** | **7** | /**sɑkɑrɑ**/ <سَكَرَ> “got drunk” | /**sɑkːɑrɑ**/ <سَكَّرَ> “closed” |
|  | **8** | /**rɑkɑzɑ**/ <رَكَزَ> “established” | /**rɑkːɑzɑ**/ <رّكَّزَ> “concentrated” |
| **/t/** | **9** | /**kɑtɑmɑ**/ <كَتَمَ> “kept” | /**kɑtːɑmɑ**/ <كَتَّمَ> “prevented” |
|  | **10** | /ʔ**ɑtɑbɑ**/ <عَتَبَ> “blamed” | /ʔ**ɑtːɑbɑ**/ <عَتَّبَ> “built a doorstep” |
| **/tˁ/** | **11** | /**fɑtˤɑrɑ**/ <فَطَرَ> “sorrowed” | /**fɑtˤːɑrɑ**/ <فَطَّرَ> “fed” |
|  | **12** | /**nɑtˤɑkɑ**/ <نَطَقَ> “pronounced” | /**nɑtˤːɑkɑ**/ <نَطَّقَ> “someone made someone else to utter” |

**Supplement S6**

| Table S6. | | *Task (3) a reading task “ortho-with-diacritics”: carrier sentences* | | |
| --- | --- | --- | --- | --- |
| **Read the following sentences:** | | | | |
|  | **Transcription** | | **Arabic sentences** | **Meaning** |
| **1** | I say /**sɑkːɑrɑ/** once again. | | **أنا أقول** سَكَّرَ **مرة أخرى.** | I say **closed** once again. |
| **2** | I say /**ʔɑbːɑdɑ/** once again. | | **أنا أقول** عَبَّدَ **مرة أخرى.** | I say **built** once again. |
| **3** | I say /**lɑbɑsɑ/** once again. | | **أنا أقول** لَبَسَ **مرة أخرى.** | I say **put on** once again. |
| **4** | I say /**ħɑdːɑθɑ/** once again. | | **أنا أقول** حَدَّثَ **مرة أخرى.** | I say **told** once again. |
| **5** | I say /**kɑtɑmɑ/** once again. | | **أنا أقول** كَتَمَ **مرة أخرى.** | I say **kept (a secret)** once again. |
| **6** | I say /**ʔɑtːɑbɑ/** once again. | | **أنا أقول** عَتَّبَ **مرة أخرى.** | I say **built a doorstep** once again. |
| **7** | I say /**qɑdːɑrɑ/** once again. | | **أنا أقول** قَدَّرَ **مرة أخرى.** | I say **estimated** once again. |
| **8** | I say /**sɑkɑrɑ/** once again. | | **أنا أقول** سَكَرَ **مرة أخرى.** | I say **got drunk** once again. |
| **9** | I say /**nɑtˤɑkɑ/** once again. | | **أنا أقول** نَطَقَ **مرة أخرى.** | I say **pronounced** once again. |
| **10** | I say /**ħɑdɑθɑ/** once again. | | **أنا أقول** حَدَثَ **مرة أخرى.** | I say **happened** once again. |
| **11** | I say /**kɑtːɑmɑ/** once again. | | **أنا أقول** كَتَّمَ **مرة أخرى.** | I say **prevented** once again. |
| **12** | I say /**fɑtˤːɑrɑ/** once again. | | **أنا أقول** فَطَّرَ **مرة أخرى.** | I say **fed** once again. |
| **13** | I say /**ʔɑbɑdɑ/** once again. | | **أنا أقول** عَبَدَ **مرة أخرى.** | I say **worshiped** once again. |
| **14** | I say /**rɑkɑzɑ/** once again. | | **أنا أقول** رَكَزَ **مرة أخرى.** | I say **established** once again. |
| **15** | I say /**fɑtˤɑrɑ/** once again. | | **أنا أقول** فَطَرَ **مرة أخرى.** | I say **sorrowed** once again. |
| **16** | I say /**sɑmːɑrɑ/** once again. | | **أنا أقول** سَمَّرَ **مرة أخرى.** | I say **burned** once again. |
| **17** | I say **/lɑbːɑsɑ/** once again. | | **أنا أقول** لَبَّسَ **مرة أخرى.** | I say **put (some clothes) on (someone)** once again. |
| **18** | I say /**ʔɑtɑbɑ/** once again. | | **أنا أقول** عَتَبَ **مرة أخرى.** | I say **blamed** once again. |
| **19** | I say /**ʃɑmːɑrɑ/** once again. | | **أنا أقول** شَمَّرَ **مرة أخرى.** | I say **rolled up** once again. |
| **20** | I say /**sɑmɑrɑ/** once again. | | **أنا أقول** سَمَرَ **مرة أخرى.** | I say **spent (his night)** once again. |
| **21** | I say /**nɑtˤːɑkɑ/** once again. | | **أنا أقول** نَطَّقَ **مرة أخرى.** | I say **made someone else to utter** once again. |
| **22** | I say **/ʃɑmɑrɑ/** once again. | | **أنا أقول** شَمَرَ **مرة أخرى.** | I say **walk proudly** once again. |
| **23** | I say /**qɑdɑrɑ/** once again. | | **أنا أقول** قَدَرَ **مرة أخرى.** | I say **was able** once again. |
| **24** | I say /**rɑkːɑzɑ/** once again. | | **أنا أقول** رَكَّزَ **مرة أخرى.** | I say **concentrated** once again. |
